# Novel *CPLANE1* c.8948dupT (p.P2984Tfs*7) variant in a child patient with Joubert syndrome

**DOI:** 10.1515/biol-2022-0542

**Published:** 2023-02-01

**Authors:** Huiping Wang, Wensha Nie, Chunxia Wang, Zuohua Wang, Yuxia Zheng

**Affiliations:** Department of Neurology, Kunming Children’s Hospital, Kunming Children’s Hospital Affiliated with Kunming Medical University, No. 288, Qianxing Road, Xishan District, Kunming 650228, China

**Keywords:** Joubert syndrome, *CPLANE1*, oculomotor apraxia, ataxia, molar tooth sign

## Abstract

Joubert syndrome (JBTS) is a class of heterogeneous ciliopathy genetically associated with *CPLANE1* mutations. The characteristics of clinical phenotypes and *CPLANE1* variants were analyzed in a 2-month-old patient. A 2-month-old patient with JBTS was diagnosed after clinical evaluation including family history, physical examination, cerebral MRI, ultrasonography imaging, VEGG, ocular fundus examination, and comprehensive blood and urine testing. Whole exome sequencing (WES) was performed to detect *CPLANE1* variants, and Sanger sequencing was used to confirm the variants. This JBTS patient presented with oculomotor apraxia, dysregulation of breathing pattern, and ataxia. MRI revealed poor continuity of cerebelli, batwing appearance, and molar tooth sign. This patient was noted with abnormal hematology, dysregulation of hepatic function, thyroid function, immunity, and renal function, and encephalopathy. *CPLANE1* (c.8948dupT (p.P2984Tfs*7) and c.247G > T (p.G83X)) variants were noticed in the patient as a pathogenic variant and caused autosomal recessive inheritance. The JBTS patient with mutations in *CPLANE1* (c.8948dupT (p.P2984Tfs*7) and c.247G > T (p.G83X)) developed JBTS phenotypes. The novel *CPLANE1* c.8948dupT (p.P2984Tfs*7) variant will assist clinicians and geneticists in reaching a precise diagnosis for JBTS.

## 1 Introduction

Joubert syndrome (JBTS) is defined as a group of ciliopathies typical of neurologic abnormalities (global developmental delay, ataxia, molar tooth sign, cerebellar vermis hypoplasia, and cerebellar vermis agenesis) and other congenital defects including oculomotor apraxia, hyperventilation or abnormal breathing pattern, and polydactyly and syndactyly. Joubert et al. first reported four French Canadian sibs diagnosed with severe neurologic disorders manifesting episodic hyperpnea, ataxia, oculomotor apraxia, and mental retardation [1]. Srour et al. subsequently depicted the molar tooth sign in JBTS patients [2]. There is growing evidence that variable multiorgan is affected in JBTS, including the retina, kidney, liver, and skeletal system [3,4,5]. Exome and Sanger sequencing has confirmed [6,7,8,9,10,11,12,13] a significant association between JBTS17 and compound heterozygous mutation in the gene encoding ciliogenesis and planar polarity effector 1 (CPLANE1; Alternative name: JBTS17) protein [2,12,14,15].

CPLANE1 gene (synonyms: *C5orf42*, *JBTS17*, *OFD6*, and *Hug*) mutations change the encoding pattern of ciliary proteins affecting ciliary functions [16]. CPLANE1 protein localizes to the ciliary transition zone, mediating the recruitment of peripheral IFT-A proteins to basal bodies, cell polarity and migration, and ciliogenesis [16]. CPLANE1 protein is necessary for the assembly of the cilia transition zone complex [16]. *CPLANE1* silence interferes with the pathfinding of commissural axons, retards neural circuit formation, and causes abnormal facial features in chicken embryos [17]. Cell-cycle-dependent proteolysis regulates localization of CPLANE1 to the mitotic kinetochore [18]. *CPLANE1* deficiency delays mitosis and chromosome misalignment, and causes defective radial migration of postmitotic cells [18].

It was determined that JBTS patients carry compound heterozygous mutations in *CPLANE1*, including missense variants (c.2876C > T, c.4006C > T, c.4067C > T, c.4690G > A), splicing mutations (c.2292-2delA, c.3921 + 1G > A, c.7400 + 1G > A, c.8471-1G), and frameshift mutations (c.230_233del, c.4804C > T, c.6407del, c.6997_6998insT, c.7477C > T), reported in the literature [2,14]. Recently, we diagnosed a child patient with JBTS. Whole exome sequencing (WES) and Sanger sequencing confirmed *CPLANE1* mutations in our JBTS patient. This novel discovery of the *CPLANE1* variants will help clinicians and geneticists to make an accurate diagnosis of JBTS.

## 2 Case presentation

The patient was normally delivered. The birth was full term. One day after birth, the patient developed respiratory distress due to choking on milk. The patient was diagnosed with neonatal respiratory distress syndrome, pneumonia, hyperbilirubinemia, myocardial damage, coagulation abnormalities, and atrial septal defect. The patient was then hospitalized for ventilator-assisted ventilation and discharged 9 days after. At 2 months of age, the patient was presented to our hospital. His parents complained that the patient appeared horizontal head tremor during wakefulness without specific inducements 9 days ago. This symptom was especially manifest when crying, while vanished when sleeping or suckling. Since the onset, the patient showed no signs of mental or motor regression and had no problems with sleeping, urination, and defecation. The patient’s parents denied the genetic history of the disease and other disorders.

Body temperature (36.7°C), heart rate (130 beats/min), breathing (32 times/min), and body weight (3.98 kg) were normal. The child patient was inclined to tilt his head to the right and look to the right and appeared horizontal head tremor without specific inducements. Ophthalmological examination revealed pupillary isocoria, equal pupils, and normal pupillary light reflex. Patient’s performance during the smooth pursuit was poor. Nystagmus was not observed. The patient was not noted to have enlarged superficial lymph nodes, cyanosis, rash, throat congestion, and inspiratory recession of the suprasternal fossa, supraclavicular fossa, and intercostal spaces. An abnormal laryngeal appearance was not observed. Coarse breath sounds were bilaterally noted by auscultation. There were no obvious pulmonary dry crackles and moist crackles. The heart pulsates powerfully with a regular rhythm and no murmur. Clinical abdominal examination showed abdominal softness, no abdominal distension, and no crying episode. Splenomegaly and hepatomegaly were not detected by abdominal palpation. Neurological examination showed no nuchal rigidity. Cranial nerve injury was not noted. The patient had grasp reflex hyperreflexia, and abdominal and patellar reflex. Babinski signs were not evident. There were no limb convulsions. High extremity muscle tensions were noted when the patient was quiet, especially after stimulation. The upper arm slightly rotated inward, and the right hand rotated clockwise with the thumb. Slightly repeated head nodding was occasionally observed. The patient was easily frightened.

**Figure 1:**
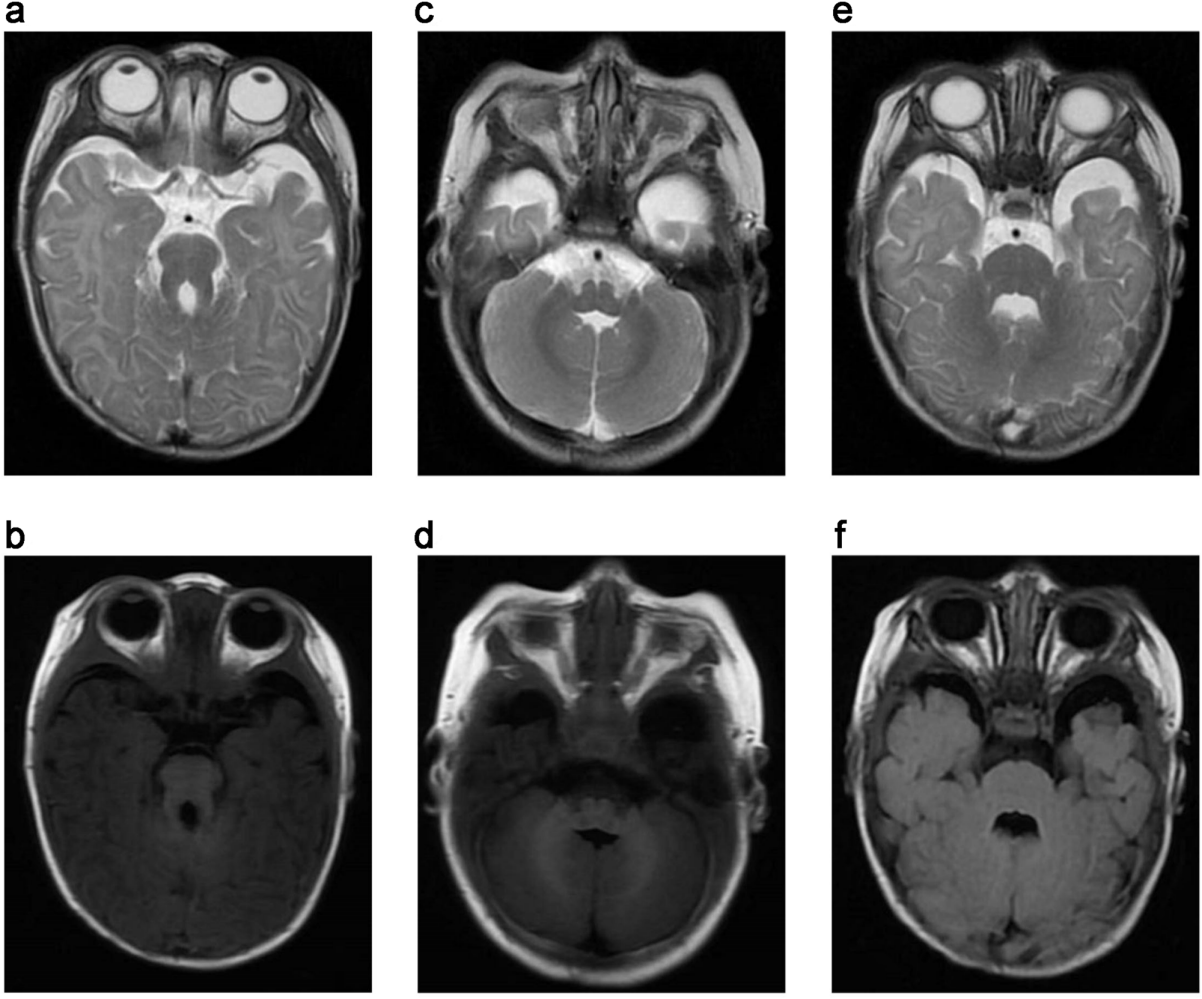
Brain MRI of the affected patient showing evidence of a molar tooth sign (a, T1WI; b, T2WI), dysplastic cerebellar vermis (c, T1WI; d, T2WI), and the typical “bat wing” morphology (e, T2WI; f, T2 fluid-attenuated inversion recovery sequence axial MRI).

Cerebral magnetic resonance imaging (MRI) examinations showed a molar tooth sign (Figure 1a and b). Figure 1c and d show the widened extracerebral spaces of bilateral temporal lobes and poor continuity of cerebellar hemispheres. The fourth ventricle gave rise to the batwing appearance (Figure 1e and f). Morphological and signaling alterations were not observed in brain parenchyma. Diffusion-weighted imaging showed no diffusion restriction. Brain ventricular system showed normal morphology. The subarachnoid space was enlarged. Cisterns, sulci, and fissures were still within the normal range. Brain midline shift was not observed. No abnormality was noted in cerebellum and brainstem. Cranial ultrasound and computed tomographic scans showed normal results. EGG showed delta activity was increased. No significant discharges were noted when the patient shook his head. The patient’s head shook vigorously, and the upper limbs stretched a little, and a small number of fast waves were seen on the synchronized EEG.

The sonograms of the liver, bile, spleen, and both kidneys showed no significant abnormalities. Cardiac ultrasound revealed a patent foramen ovale that has been considered a possible risk factor for ischemic stroke. Electrocardiograph suggested sinus tachycardia. Abnormalities in blood and urine chemistries were measured. Table A1 summarizes the abnormal results. Blood investigation of lymphocyte, neutrophil, hemoglobin, and platelet was abnormal. The patient had hepatic problems with increased levels of ALT, TG, ALP, DBil, total bile acid, and GOT. Routine examination revealed abnormalities in thyroid function, immune function, and renal function. This patient exhibited signs of hyoxemia. Elevated plasma ammonia and cerebrospinal fluid (CSF) chloride showed indications of encephalopathy. Hypersensitivity examination of carbamazepine and oxcarbazepine HLA-B was negative in carbamazepine- or oxcarbazepine- induced hypersensitivity. Hepatotoxicity of sodium valproate by genetic variants of *POLG* was excluded.

**Figure 2:**
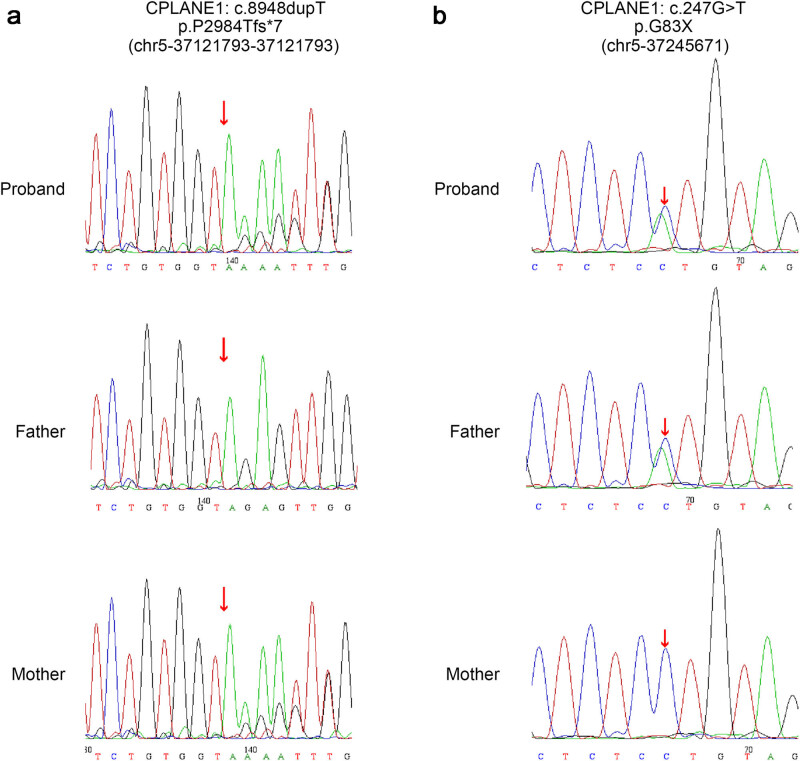
Sanger sequencing suggesting the proband and his mother were heterozygous for CPLANE1 c.8948dupT (p.P2984Tfs*7) variant (a), and the proband and his father were heterozygous for CPLANE1 c.247 G > T (p.G83X) variant (b).

**Figure 3:**
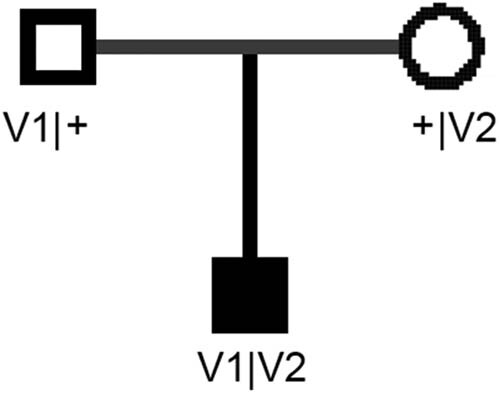
A pedigree of the family studied in this study. V1, c.8948dupT (p.P2984Tfs*7); V2, c.247 G > T (p.G83X). Circles, females; Squares, males. The black square presented the proband.

**Figure 4:**
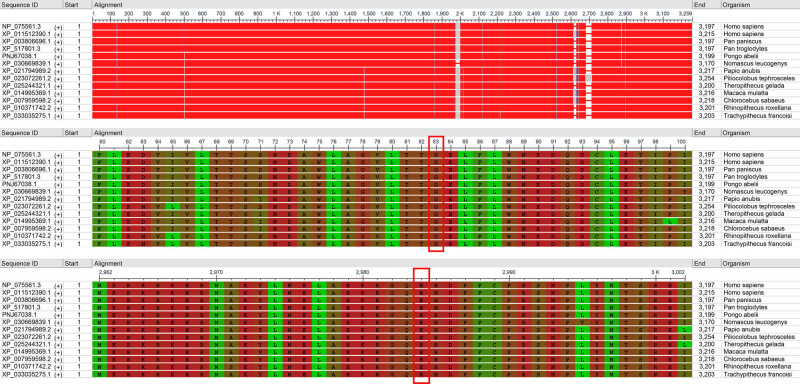
Multiple sequences alignment depiction of CPLANE1 gene. The red boxes represent the mutant sites p.G83X and p.P2984Tfs*7.


WES and Sanger sequencing detected two compound heterozygous variants of *CPLANE1* (NM_023073): c.8948dupT (p.P2984Tfs*7) (Figure 2a) and c.247G > T (p.G83X) (Figure 2b). No other potentially pathogenic variants in other genes associated with JBTS were excavated in this study. c.8948dupT in exon48 caused the frameshift in the 8948th base and a substitution of proline by threonine at the 2984th amino acid of the encoded protein. According to the ACMG, *CPLANE1* c.8948dupT variant resulted in the loss of function of *CPLANE1* (PVS1); this variant was presented with a low frequency in the general population (PM2); *CPLANE1* c.8948dupT variant was defined as a pathogenic variant and caused autosomal recessive inheritance (PM3); the patient’s phenotype was highly specific to a basic disease with a single genetic inheritance (PM4). This variant has not been recorded in the ClinVar database. c.247G > T in exon4 resulted in a change of G to T that elicited a substitution of glycine at the 83rd amino acid (p.G83X). The ClinVar database indicated that this variant is pathogenic and caused inborn genetic diseases (PS1). Based on the ACMG guidelines, *CPLANE1* c.247G > T was a pathogenic variant (PVS1 + PS1 + PM2 + PM3 + PP4). Sanger sequencing confirmed that c.8948dupT (p.P2984Tfs*7) was of maternal origin, and c.247G > T (p.G83X) was paternally inherited, which was depicted in the pedigrees (Figure 3). Both *CPLANE1* c.8948dupT and c.247G > T variants occurred within the conserved protein sequences (Figure 4). Conserved domain analysis revealed that the function of JBTS-associated domain is still unconfirmed [19]. However, mutations of *CPLANE1* lead to JBTS, which is related to ciliogenesis [16]. Figure 4 shows the alignment of multiple *CPLANE1* sequences. It was found that the two variants c.8948dupT (p.P2984Tfs*7) and c.247G > T (p.G83X) are highly conserved across the alignment sequences from the indicated species.

**Informed consent:** Informed consent has been obtained from all individuals included in this study.
**Ethical approval:** The research related to human use has been complied with all the relevant national regulations, institutional policies and in accordance with the tenets of the Helsinki Declaration, and has been approved by the Research Ethics Committee of Kunming Children’s Hospital (No. 2021-03-050-K01).

## 3 Discussion

JBTS is typically characterized by cerebellar vermis hypoplasia, developmental delays, and irregular breathing pattern [1]. Retinal degeneration and renal anomalies have also been included in JBTS17 patients [20,21]. *CPLANE1* has been predicted to encode a protein with transmembrane domains and coiled-coil domains, which may be involved in ciliogenesis and mitotic process [22,23]. There has been accumulated evidence that numerous *CPLANE1* mutations are pathogenic variants in patients with JBTS [24,25,26,27]. In this report, we describe a 2-month-old patient diagnosed with JBTS. Physical examination, laboratory data, and MRI findings revealed the diagnosis of JBTS. The diagnosis was confirmed after the detection of *CPLANE1* c.8948dupT (p.P2984Tfs*7) and c.247G > T (p.G83X) variants by WES and Sanger sequencing.

The diagnosis of JBTS is mainly on the hallmark of molar tooth signs in brain imaging and features of hypotonia and developmental delays. These classic symptoms were generally accompanied by breathing abnormalities, truncal ataxia, cognitive abilities, retinal dystrophy, hepatic disease, ocular colobomas, occipital encephalocele, and polydactyly [3,5,20,28,29,30,31]. The clinical characteristics of our patient included typical developmental delay, oculomotor apraxia, breathing abnormalities, truncal ataxia, cerebellar vermis hypoplasia, dysregulation of hepatic function, thyroid function, immunity, and renal function, and encephalopathy. The characteristic clinical features and MRI findings have been described in previously reported cases [3,5,20,28,29,30,31], which are the fundamental criterion in the diagnosis of JBTS.

The main oculomotor abnormalities in patients with JBTS include ocular motor apraxia, nystagmus, and strabismus [28,29]. However, our patient presented with strabismus but no nystagmus. Short alternate episodes of apnea and tachypnea are commonly observed [30], which are also noticed in our case. A spectrum of organ defects is demonstrated in JBTS patients commonly retinal defects, renal defects, and congenital liver fibrosis [3,5,20], which is consistent with our case. Other rarely observed features such as midline oral and facial defects, Hirschsprung disease, skeletal dysplasia, severe scoliosis, situs inversus, congenital heart malformations, and optic nerve colobomas were not observed in our patient. Based on the findings of physical examination, laboratory data, and MRI, this patient was presumptively diagnosed with JBTS.

As a genetically heterogeneous primary ciliopathy, JBTS has been detected with pathogenic variants in numerous genes including *CPLANE1* [29,32,33]. *CPLANE1*-encoded protein is involved in ciliogenesis, which affects cell polarity and directional cell migration [16]. Cilium assembly or disassembly markedly affects extracellular signals mediating growth and developmental syndromes related to multiple organs and systems such as brain, retina, skeletal system, kidney, liver, skeletal system, and pancreas [34]. It has been confirmed that the knockdown of *CPLANE1* caused ciliopathy-related development defects in neural tube closure, Hedgehog signaling, and left-right patterning in Xenopus embryos [35]. The *CPLANE1* variants recorded in the ClinVar are classified into five types: 37 frameshift, 153 missense, 28 nonsense, 13 splice site, and 25 untranslated region types. We found a novel *CPLANE1* variant c.8948dupT, which has not been documented in the ClinVar and literatures. Protein structure analysis revealed that c.8948dupT and c.247G > T variants change the highly conserved regions, suggesting that the clinical phenotypes may be caused by the two variants. Additionally, couples with a JBTS child have a 25% risk of recurrence. Hence, prenatal diagnosis through chorionic villus sampling at approximately 11 weeks of gestation is necessary and feasible only for proband family with *CPLANE1* variants.

Respiratory and feeding problems should be particularly focused to avoid hypotonia and respiratory abnormalities. Intensive monitoring is required to manage renal failure and delay complications. Specific follow-up must be scheduled to treat congestive heart failure. Breathing dysregulation is especially associated with prognosis. Assisted ventilation may reduce the recurrent probabilities of life-threatening prolonged apneas. Dystonia was treated by oral trihexyphenidyl (0.25 mg/time, twice a day during the first to third day; 0.5 mg/time, twice a day from the 4th day). After one week of medication, the patient’s tremor gradually subsided, and the head tremor disappeared 1 month after discharge. Compound glycyrrhizin tablets were administered twice a day, and the patient’s liver function improved 1 month after discharge. However, the patient’s thumbs often retracted inward, and his movements were usually awkward and inflexible, especially in the hands. The patient still has difficulty placing two square toys together.

## 4 Conclusions

In this study, we diagnosed an infant patient with JBTS typical of hypoplasia of the cerebellar vermis, irregular breathing pattern, retinal degeneration, and renal anomalies. WES and Sanger sequencing identified 2 mutations in *CPLANE1* (c.8948dupT (p.P2984Tfs*7) and c.247G > T (p.G83X)) in an infant patient with JBTS. *CPLANE1* c.8948dupT (p.P2984Tfs*7) is a newly reported variant that is not recorded in the ClinVar database or documented in the literature. Further, oral trihexyphenidyl alleviated dystonia and the compound glycyrrhizin tablets improved liver function. Regular follow-up is necessary. This novel finding in *CPLANE1* variants will assist clinicians and geneticists in reaching a precise diagnosis for JBTS.

## Appendix

**Table A1:** Laboratory findings of the patient with JBTS

	Patient	Normal range		Patient	Normal range
Whole blood analysis			Renal function^a^		
Lymphocyte (×10^9^/L)	4.02	1.0–3.0	Creatinine (μmol/L)	25.31	27–62
Lymphocytes (%)	79.00	20–40	Adrenal function^a^		
Neutrophil (×10^9^/L)	0.55	1.8–6.4	Cortisol (nmol/L)	98.72	66–630
Neutrophil (%)	10.80	50–70	ACTH (pg/mL)	23.36	7.2–63.6
Hemoglobin (g/L)	107.00	110–160	Arterial blood gas analysis^c^		
Hematocrit (%)	30.30	37–50	Ca^2+^ (mmol/L)	1.40	1.15–1.32
Platelet (×10^9^/L)	449.00	100–300	pH value	7.34	7.35–7.45
Thrombocytocrit	0.44	0.108–0.282	PCO_2_ (mmHg)	43	35–45
Platelet distribution width (fL)	11.10	15.5–18.1	PO_2_ (mmHg)	30	80–100
Hepatic function^a^			Bicarbonate (mmol/L)	23.2	—
Ceruloplasmin (g/L)	0.22	0.2–0.6	Base excess (mmol/L)	–2.5	−3 to +3
ALT (U/L)	125	0–40	Oxygen saturation (%)	57.0	95.00–99.00
TG (U/L)	57	0–50	Lactate (mmol/L)	3.2	0.5–2.2
ALP (U/L)	455	147.7–309.3	Encephalopathy		
Total protein (g/L)	57.9	60–80	Serum blood glucose (mmol/L)^d^	5.4	3.9–5.8
DBil (μmol/L)	5.1	0–3.4	Plasma ammonia (μmol/L)	51.8	10–50
Total bile acid (μmol/L)	17.6	0–10	CSF color	Colorless/Transparent	Colorless/transparent
GOT (U/L)	121	0–40	CSF WBC (×10^9^/L)	2 × 10^6^/L	—
Bone development^b^			CSF chloride (mmol/L)	123.6	111–123
PTH (pmol/L)	2.40	1.6–6.9	CSF glucose (mmol/L)	3.04	2.8–4.5
25–OH-D (nmol/L)	99.28	75–250	CSF protein (g/L)	0.12	0–0.4
Anemia^a^			Encephalitis		
Serum folate (ng/mL)	40	7.1–23	*Candida albicans* ^e^	Negative	—
Thyroid function^a^			Cryptococcus^f^	Negative	—
TSH (mIU/L)	4.85	0.27–4.2	Acid-fast bacilli^g^	Negative	—
FT4 (pmol/L)	13.28	14–23	Parasites and viruses		
Immune function^a^			Toxoplasma IgM^b^	Positive	—
Globulin (g/L)	12.30	20–40	Cytomegalovirus (IU/mL)^e^	21,400	—
IgM (g/L)	0.25	0.06–0.21	CMV IgM antibody	Positive	—
IgA (g/L)	0.04	0.05–0.34	HBsAb	Positive	—
C3 (g/L)	0.652	0.67–1.24	Anti-HBs	Positive	—
C4 (g/L)	0.057	0.09–0.30			

^a^Serum samples. ^b^Venous blood. ^c^Arterial blood. ^d^Detected by the point-of-care testing before lumbar puncture. ^e^The detection was performed 4 days after the culture of encephalitis. ^f^India ink staining. ^g^Anti-acid bacteria staining. GOT, glutamic oxaloacetic transaminase; IgG, immune globulin; Ig, immunoglobulin; ACTH, adrenocorticotropic hormone; PTH, parathyroid hormone; 25-OH-D, 25-hydroxy vitamin D; TSH, thyroid stimulating hormone; FT4, free thyroxine; CSF, cerebrospinal fluid; WBC, white blood cells; RBC, red blood cell; HBsAb, hepatitis B surface antibody; CMV, cytomegalovirus; Anti-HBs, hepatitis B surface antibody; ALT, alanine transaminase; TG, transglutaminase; ALP, alkaline phosphatase; DBil, direct bilirubin; JBTS, Joubert syndrome.
